# Influenza Epidemic in South Africa

**Published:** 1919-01-11

**Authors:** 


### INFLUENZA EPIDEMIC IN SOUTH AFRICA.

### FIFTY THOUSAND DEATHS.

A South African correspondent, writing on October 29, says:—The epidemic in South Africa of what is known as Spanish Influenza has during its month's course had a result in glutted mortality of some fifty thousand lives. The scourge is now gradually subsiding in the large towns, but is still active in villages and country districts where the untutored native is a great factor in its propagation. It is a big toll of human life in a brief month, and is about twice the number of fatalities suffered by South African troops during the Great War. Roughly, ten thousand of the fifty thousand deaths are those of Europeans. As in the Moloch of war, the strong man in the prime of health has been a favourite victim of La Grippe.

There are many theories as to the origin of the epidemic. It has certainly been introduced from Europe. Some attribute it to returning South African Labour battalions, others to trench fever, German gas, and to deliberate Hun propaganda. But it might easily enough have been introduced quite innocently from England, where influenza had been developing for some months previously. South Africa was fortunate enough to escape invasion from the La Grippe scourge in England thirty years ago, and that it was very fortunate in having done so the present widespread epidemic shows. The facility of transmitting infection by our omni-present native population is the great danger.

One result of the epidemic is that quarantine has been imposed in Australia on South African vessels arriving at their ports of entry.

Inoculation has been successful at Capetown and in other towns, where it has been done on a pretty large scale. Everywhere simple safeguards have been in vogue, such as gargling the mouth and nostrils with salt water, snuffing and phenol. The resultant pneumonia has been the prevailing cause of mortality. The towns that have suffered most from the scourge are Capetown, Kimberley, Johannesburg, and Bloemfontein. There have been thirty thousand cases in Durban, which has a population of 84,000—half white and half coloured —but they have been mild in form, and there have been comparatively few deaths. In most of the towns drastic precautionary measures have been adopted, including closing of schools, places of entertainment and of worship, prohibition of public meetings and other public gatherings, closing of market halls and auction rooms. Shops were in some towns closed, and in most towns are still doing business in restricted hours. At the present time the epidemic is sensibly subsiding in the towns, though not in the villages and in the country districts generally. At, for instance, the Wallsend Colliery, Natal, half of the employees are at the present time down with influenza. The appeal of the Union Health Department for doctors and nurses has been as well responded to as possible. A hundred Durban ladies have left there for various centres to assist in nursing.

One case shows the widespread influence of the disease. A Mr. Herbert Wardle, aged 22, died at the Pietermaritzburg Sanatorium two days ago. Unknown to him a sister of his, a nurse, has died at Wynberg, another sister at Aliwal North, and a brother at Queenstown, the home of the family. All have died from influenza.

Capetown was the scene of the initial outbreak about a month ago. From the first to the twenty-fifth of this month there have been 7,400 deaths from the epidemic in the Cape Peninsula, and one fourth of them were European. As in other parts of the Union, the railway, postal, and tramway staffs were greatly depleted, and their services have been necessarily curtailed. At one time Capetown was described as being, not only figuratively but literally, a city of the dead. Most of the shops were shut down, those open were doing practically no business, and the streets were deserted except at the railway station, where crowds waited, watching for funerals. The normal number of funerals at Capetown is ten a day. During the height of the disease there were as many as three hundred daily. There were 1,650 free coffins issued at the City Hall. And there are over a thousand children now left destitute owing to the passing of their parents.

Johannesburg has lost 3,000 out of a European population of 132,000, and has therefore emerged from the fire comparatively favourably. There were many thousands of cases of influenza among the natives on the mines, and at one time there were no less than 17,000 cases in hospital. All schools were closed, the Town Council voted £10,000, and provided free treatment and food for the poor. The schools were turned into hospitals. The fine spirit of co-operation manifested by the citizens of the Golden City had a great deal to do with the successful coping with the disease. A sad fatality, resulting in a loss of nineteen lives, occurred at the Driefontein Mine owing to the Europa winding-engine driver being suddenly attacked by influenza.

No town suffered more severely than Kimberley, the diamond centre. The death-roll amounted to 4,405, of which number 536 were European. More than half of the deaths occurred in the mine compounds. The death rate was 10 per cent. The entire business life of the town was suspended for a time. It had indeed the appearance of being a huge hospital camp. Normal business was resumed yesterday. Col. Oronstein, the Panama expert, built up a thorough organisation for combating the disease.

For a small town, Bloemfontein's death-roll was a remarkably heavy one, numbering 1,218, embracing 370 Europeans and 848 natives. The disease was at its worst in the middle of the month.

**Influenza Epidemic in South Africa**—(*continued*). Tuesday, October 15, recorded 118 deaths, October 16 105, October 17 112, and October 18 135. Of these 132 were deaths of Europeans and 337 natives.

Pretoria had 800 deaths in fifteen days. All entertainments were closed down, and there was a scarcity of doctors and nurses.

Drastic precautionary measures were taken at Pietermaritzburg, with the result that the mortality has been light; as it has also been at Durban. At Port Elizabeth there have been 746 deaths, of which 27 per cent. were those of Europeans. Over 200 deaths have occurred at Port Alfred, and 161 at East London, including 35 Europeans.

Potchefstroom, the great military centre in the Transvaal, had 220 deaths including 49 European cases in camp and 50 in town. One of the victims was the acting district surgeon. At Harrismith there have been 24 deaths of Europeans. Here two Durban medical men and nine nurses are helping. Among the deaths are the Chairman of the Health Board and the wife of the Magistrate. Herschel, in East Griqualand, reports 300 deaths, Carnarvon 83 white deaths, Prieska 30 European and 50 native deaths—all small places.

There are 625 native boarders at the Lovedale Training College, Cape Province. Of these 555 took the disease; but there have been only five deaths.

The disease has spread widely in the Bechuanaland Protectorate. Three hundred natives died from it at Kamoutsa. There are about 8,000 at Canga, which is 90 per cent. of the population. Khama's capital is affected. At Mafeking the eleven European deaths include the Medical Officer of Health, Dr. H. A. Dean, Nurse Zurich, Mrs. Proctor, a voluntary nurse, and the district superintendent of railways. At Maclear, East Griqualand, the only medical man succumbed to the scourge, which is especially severe there and at Matatiele. At Ladysmith the European deaths number 30

There are 39,000 employees in the South African Railway Department. Of these 15,660 have been off duty owing to the epidemic, including 8,369 Europeans. Railway traffic has consequently been curtailed. It would probably have been better if all native passenger traffic had been suspended, as the conveyance of natives and also of Indians is a fertile source of infection.

The epidemic affected the gold production on the Rand during September to the extent of 3,200 ounces, or a loss of £136,000 in a total of £3,000,000 for the month.

Outside of the Union the position in Basutoland is regarded as rather serious. In Rhodesia, Salisbury, the capital, last week has been one of great anxiety. European cases run into hundreds, and there have been 36 deaths. There are thousands of native cases and 200 deaths. Three of the five medical men are down with the disease, but volunteers are giving splendid help.

Among prominent men who have succumbed to the disease in South Africa have been Captain Tom Maginess, M.C., formerly Labour member for a Capetown constituency, Mr. H. C. Becker, Government Party Whip, and M.L.A. for Ladysmith, Hon. Mrs. Cloete, and Drs. Spaulding, Wille, J. A. Thwaites, L. A. W. Beck, A. S. Kung, R. Borcherds, Lt.-Col. Van Zyl; also several nurses.

